# Néphrocalcinose compliquant une miliaire tuberculeuse chez un nourrisson

**DOI:** 10.11604/pamj.2015.20.425.6542

**Published:** 2015-04-29

**Authors:** Jaouad El Maghraoui, Fatima Zahrae Souilmi, Mohamed Hbibi, Tarik Sqalli Houssaini, Mustapha Hida

**Affiliations:** 1 Service de Néphrologie, CHU Hassan II, Fès, Maroc; 2 Service de Pédiatrie, CHU Hassan II, Fès, Maroc

**Keywords:** Hypercalcémie, intoxication à la vitamine D, néphrocalcinose, tuberculose, Hypercalcemia, vitamin D intoxication, nephrocalcinosis, tuberculosis

## Abstract

La néphrocalcinose se définit par la présence anormale dans le parenchyme rénal de dépôts calciques pouvant résulter de différentes affections. Nous rapportons le cas d'un nourrisson de 7 mois chez qui nous avons diagnostiqué une hypercalcémie compliquée par une néphrocalcinose secondaire à une tuberculose miliaire aggravée par l'intoxication à la vitamine D améliorée sous hyperhydratation, furosémide et glucocorticoïdes. A travers ce travail, nous insistons sur l'intérêt de la recherche d'une hypercalcémie devant toute infection tuberculeuse, de la prise en charge rapide et adaptée sans oublié d'interrompre la supplémentation en vitamine D.

## Introduction

La néphrocalcinose se définit par la présence anormale dans le parenchyme rénal de dépôts calciques pouvant résulter de différentes affections. Les étiologies classiques de l'hypercalcémie chez l'enfant sont l'hyperparathyroïdie, l'hypercalcémie hypocalciurique familiale, l'intoxication à la vitamine D, l'insuffisance surrénalienne et le syndrome de Williams et Beuren [1–3]. L'hypercalcémie peut être associée à des granulomatoses comme la sarcoïdose [4] et la tuberculose [5–7], mais reste rare. Le mécanisme est une production inappropriée de la vitamine D3 par les monocytes activés [8]. L'incidence exacte de l'hypercalcémie en cas de tuberculose est inconnue. Nous rapportons le cas d'un nourrisson de 7 mois qui présente une miliaire tuberculeuse avec une hypercalcémie sévère compliquée d'une néphrocalcinose.

## Patient et observation

**Figure 1:**
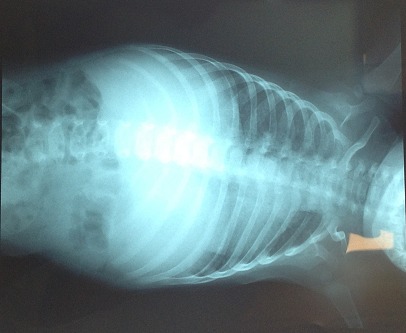
Radiographie thoracique objectivant des micronodules diffuse sur les deux champs pulmonaires évocateurs d'une miliaire tuberculeuse au cours de la première hospitalization

**Figure 2:**
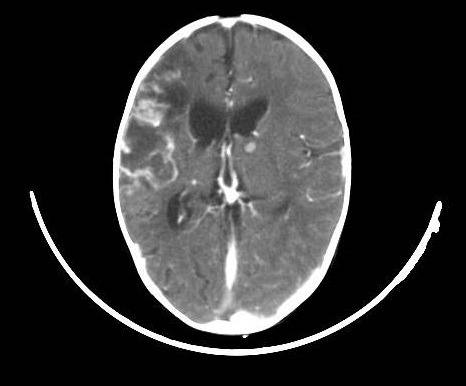
TDM cérébrale objectivant une prise de contraste nodulaire diffuse sus et sous tentorielle millimétriques (4 mm) sans effet de masse sur les structures avoisinantes, en rapport avec une origine tuberculeus

**Figure 3:**
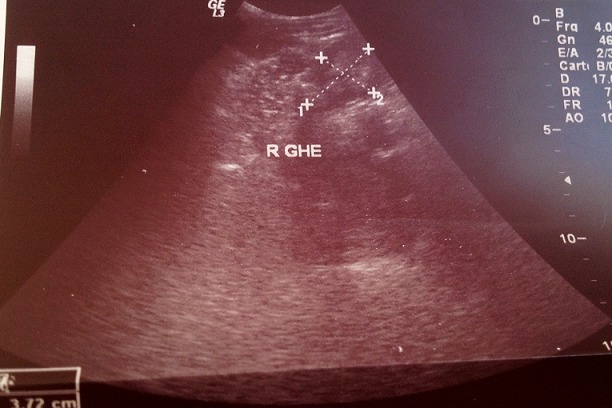
Echographie du rein gauche du nourrisson normale au cours de la première hospitalisation (27/08/2014)

**Figure 4:**
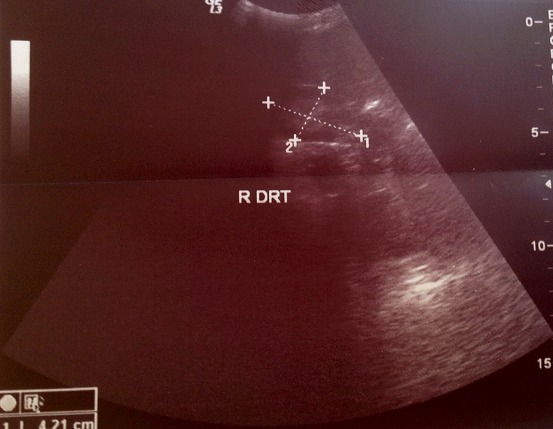
Echographie du rein droit du nourrisson normale au cours de la première hospitalisation (27/08/2014)

Il s'agit d'un nourrisson de 7 mois, ayant une notion de contage tuberculeux (la maman et la grand-mère) et chez qui nous avons diagnostiqué une miliaire tuberculeuse avec localisation neuroméningée. Suite à une symptomatologie faite de fièvre prolongée, le nourrisson a été hospitalisé le 25/07/2014 (première hospitalisation) au service de pédiatrie, l'interrogatoire a objectivé une notion de somnolence, fixité de regard sueurs et refus de téter. L'examen clinique du nourrisson a objectivé une fièvre à 40^°^C, hypotonie, hémiparésie gauche avec absence de poursuite oculaire, une hépatomégalie (flèche hépatique = 7 cm), et une splénomégalie. Le tout évoluant dans un contexte d'altération de l’état général. Une ponction lombaire réalisée a objectivé (glucorachie: 0,54g/l, protéinorachie: 0,43g/l, culture: bacilles de Koch). Une radiographie thoracique réalisée a objectivé des micronodules diffus sur les deux champs pulmonaires évocateurs d'une miliaire tuberculeuse (Figure 1). La tomodensitométrie (TDM) cérébrale a objectivé une localisation neuroméningée de la tuberculose (Figure 2). L’échographie rénale au cours de cette hospitalisation n'a pas objectivé une néphrocalcinose (Figure 3 et Figure 4). Le nourrisson était traité par le protocole RHZE pendant deux mois, et RH seul durant sept mois: Rifampicine 150mg (1/2cp par jour), Isoniazide 50mg (3/4cp par jour), Pyrazinamide 400mg (1/2cp par jour), Ethambutol 400mg (1/4cp par jour). L’évolution clinique a été bonne: nourrisson conscient, hypotonique, réactif, fontanelle antérieure normo tendue, tète bien, mais avec une persistance de l'hémiparésie gauche et stable sur le plan hémodynamique et respiratoire.

**Figure 5:**
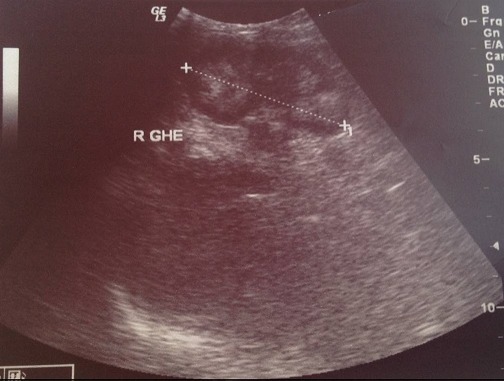
Echographie du rein gauche du nourrisson objectivant une néphrocalcinose au cours de la deuxième hospitalisation (23/09/2014)

**Figure 6:**
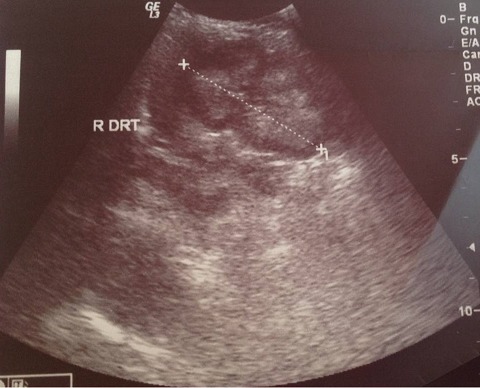
Echographie du rein droit du nourrisson objectivant une néphrocalcinose bilatérale au cours de la deuxième hospitalisation (23/09/2014)

Au cours de cette hospitalisation le patient avait une calcémie limite supérieure à 105 mg/l, 25-OH-vitamine D3 à 26,5 ng/l, protéine C réactif à 198mg/l, globules blanc à 15800/mm^3^, polynucléaires neutrophiles à 11408/mm^3^, lymphocytes à 3176/mm^3^, plaquettes à 474000/mm^3^. Quiz jours après sa sortie, le nourrisson a reçu au centre de santé une supplémentation par la vitamine D (une ampoule de stérogyl 600000UI) ce qui a aggravé son hypercalcémie à 183 mg/l. Le patient a présenté une déshydratation avec hypotonie et des vomissements, indiquant son hospitalisation le 23/09/2014. A l'admission le bilan biologique était: l'hémoglobine à 9,6g/dl, plaquettes à 382000/mm^3^, globules blancs à 11540/ mm^3^, hématocrite à 26,9%, la protéine C réactive à 4 mg / dl, l'urée sanguine 0,29 mg / l, la créatinine sérique 5 mg/ l, calcium total à 183 mg/l, phosphore à 45 mg/l, 25-OH-vitamine D3 à 160 ng/l, parathyroïde hormone (PTH) à 4.6 pg/ml. L'analyse des urines a montré une hyper calciurie des 24h à 70mg/kg/24h, une protéinurie des 24h à 10mg/kg/24h. L'examen d’échocardiogramme était réalisé à la recherche des signes électriques d'hypercalcémie, ainsi que dans le cadre du bilan étiologique du syndrome de Williams a revenu normal. Une échographie rénale réalisée a objectivé une néphrocalcinose bilatérale (Figure 5 et Figure 6) qui n’était pas présente dans la première hospitalisation. Notre patient a été mis sous hyperhydratation intraveineuse, furosémide et glucocorticoïdes. L’évolution était favorable sur le plan clinique et biologique. A sa sortie: le calcium total à 90 mg/l, le 25-OH-vitamine D3 à 28 ng/l, PTH à 20 pg/ml.

## Discussion

L'Organisation mondiale de la Santé (OMS) a signalé que 8 millions de personnes développent la tuberculose chaque année et près de 2 millions de personnes meurent à cause de cette maladie contagieuse [9]. La négligence et l'ignorance générale de la tuberculose depuis deux décennies a conduit à une augmentation de son incidence après une baisse régulière au cours du siècle dernier. Face à cette situation, l'OMS a reconnu la tuberculose comme un problème majeur de santé publique et revigoré un challenge particulièrement autour de la prévention, le diagnostic et le traitement de la tuberculose [10]. L'hypercalcémie chez l'enfant se présente par des signes neurologiques, gastro-intestinaux et rénaux tels que les vomissements, la constipation, la polyurie et la polydipsie [1]. Si cette hypercalcémie n'est pas traitée correctement des calcifications au niveau des tissus mous peuvent apparaitre dans n'importe quelle partie du corps tels que la néphrocalcinose, les calcifications ganglionnaire et les kératopathies. Dans notre cas le nourrisson a été admis pour une fièvre prolongée, sueurs et altération de l’état général avec une calcémie à limite supérieure. Hyperparathyroïdie est l'une des causes les plus fréquentes de l'hypercalcémie chez les adultes, mais il est rare chez les nouveau-nés et les enfants. L'hyperparathyroïdie est diagnostiquée lorsque l'hypercalcémie est accompagnée par des niveaux élevés de PTH [1]. Dans notre cas, le taux de PTH est normal donc l'hyperparathyroïdie était exclu. L'acidose tubulaire rénale a été exclue vu que l'analyse des gaz du sang a revenu normale dans notre cas [7].

L'hypercalcémie sévère est vue dans le syndrome de Williams. Ce syndrome est caractérisé par le visage d'elfe, un retard mental et sténose aortique supra valvulaire. Autres manifestations cliniques incluent des anomalies des dents, faible poids de naissance, petite taille et microcéphalie [3]. Dans notre cas, il n'y a pas de dysmorphies de syndrome de Williams et l'examen d’échocardiogramme était normal. L'intoxication à la vitamine D est évoqué en cas d'hypercalcémie associée à une dose élevée de 25 -OH- vitamine D3 et un taux de PTH normal [2]. Notre patient avait un taux de vitamine D normal à l'admission mais il a reçu à sa sortie de l'hôpital au centre de santé une supplémentation en vitamine D (une ampoule de stérogyl 600000 UI). Les pathologies granulomatoses telles que la tuberculose, la sarcoïdose [4] et la lèpre peuvent provoquer une hypercalcémie. Quelques études ont objectivé l'association de l'hypercalcémie et la tuberculose dans l'enfance [5–7]. Le mécanisme est une production inappropriée de la vitamine D3 par les monocytes activé qui est responsable de l'hypercalcémie [8]. Un diagnostic précoce est très important dans la tuberculose pour prévenir la propagation de l'organisme et la diffusion de la maladie [11, 12]. Dans notre cas l'hypercalcémie était due d'abord à la miliaire tuberculeuse [5, 7, 11] et aggravée par la prise de la vitamine D qui n’était pas recommandée dans ce cas. Le traitement fait appel à une hyperhydratation qui augmente l'excrétion du calcium avec les diurétiques de l'anse. En cas de persistance de l'hypercalcémie les glucocorticoïdes, la calcitonine ou les biphosphanates peuvent être nécessaires ainsi que la dialyse dans les formes sévères [1, 2, 12]. Notre patient a été amélioré sous hyperhydratation intraveineuse, furosémide et glucocorticoïdes.

## Conclusion

A travers ce travail, nous insistons sur l'intérêt de la recherche d'une hypercalcémie devant toute infection tuberculeuse surtout sévère chez le nourrisson, de la prise en charge rapide et adaptée sans oublié d'interrompre la supplémentation en vitamine D.
